# Automated Annotation of Pain Chronicity in Patients With Back Pain by Using Electronic Health Records: Retrospective Study

**DOI:** 10.2196/63198

**Published:** 2026-03-05

**Authors:** Simran Ajay Kanal, Jeannie Bailey, Jeffrey Lotz, Aaron Scheffler, Thomas Peterson

**Affiliations:** 1 Department of Epidemiology and Biostatistics University of California, San Francisco San Francisco, CA United States; 2 Department of Orthopedic Surgery University of California, San Francisco San Francisco, CA United States; 3 Bakar Computational Health Sciences Institute University of California, San Francisco San Francisco, CA United States

**Keywords:** chronic back pain, phenotyping, machine learning, electronic health records, prediction, algorithms, medical informatics, time series analysis, longitudinal data, clinical decision support, machine annotation

## Abstract

**Background:**

Chronic back pain is a severe health condition with underlying biopsychosocial factors that make diagnosis difficult, and pain chronicity has been shown to be an important variable for studying patient outcomes. Due to the absence of standardized criteria, pain chronicity needs to be manually annotated by clinicians in electronic health records (EHRs), which is not only time consuming but also has the potential to introduce variability in analysis and interpretation among practitioners.

**Objective:**

Pain chronicity is not typically recorded in EHRs and currently needs to be manually annotated by experts. Using a dataset from an interdisciplinary spine clinic consisting of 386 patients manually annotated for pain chronicity by clinical experts, this study has two objectives: (1) to examine the relationship between expert-annotated chronicity and social determinant variables present in EHRs and (2) to evaluate the feasibility of extracting pain chronicity from the EHR without expert annotation.

**Methods:**

We used a supervised machine learning model, specifically univariate regression, to examine associations between clinician-annotated pain chronicity values and the structured variables present in EHRs. Next, we trained a random forest model to predict pain chronicity by using both structured and unstructured data extracted by clinical Text Analysis and Knowledge Extraction System, a natural language processing (NLP) tool. The features extracted included clinical keywords; duration of pain reported; and the International Classification of Diseases, Tenth Revision codes. The model was assessed using the Pearson correlation coefficient and mean absolute error (MAE).

**Results:**

The study analyzed 386 patients (mean age 60.2 years, SD 16.1 years and median age 62.0 years, IQR 48.8-72.0 years) from the San Francisco Bay Area, with 62.7% (n=242) identifying as women. Our univariate regression analysis identified structured EHR variables associated with pain chronicity, which include pain severity before the last visit (*P*=.006), number of imaging orders (*P*=.006), number of visits to the neurology department (*P*=.01), and Medi-Cal insurance coverage (*P*=.03). Our random forest model using structured data showed a strong correlation of 0.887 (*P*<.001) with an MAE of 18.45 between predicted and observed chronicity, whereas our model that used the NLP tool to extract information from unstructured clinical notes and structured data showed a slightly higher correlation of 0.968 (*P*<.001) with an MAE of 10.87 between predicted and observed chronicity.

**Conclusions:**

Our study indicates that pain chronicity from EHR data could be used to study more topics on larger datasets in the future without the need for manual annotation and that using NLP tools to automate prediction is feasible.

## Introduction

While there is no singular cause, chronic back pain often stems from conditions such as sciatica or spinal stenosis [1]. Chronic back pain is already the most prevalent source of chronic pain, which cost the United States an estimated US$134.5 billion in 2016 alone, and is projected to increase by 36.4% in total cases globally by 2050 [2-4]. A characteristic of back pain is that it is often subjective in nature [5,6]. Individuals who experience pain for a prolonged period are considered to have chronic back pain. Pain is influenced by a complex mixture of biopsychosocial factors [7], and there has been increased interest in understanding how pain negatively affects an individual’s objective function. However, much work needs to be done to better understand the multifaceted approach required for treating back pain that considers the complex interplay between biological, psychological, and social factors.

Recent wide-scale adoption of electronic health records (EHRs) indicates that the health care field is evolving toward large databases of patient information. EHRs make the entry, storage, and sharing of patient information among the multiple health care providers seamless. The adoption of EHR systems opens new avenues for research and innovation in health care. As clinicians use EHR systems to document findings, this documentation provides an opportunity to study recorded data and develop automated systems for extracting information from the EHR. The automation of data extraction processes from EHRs can significantly alleviate the burden on clinicians, who would otherwise spend countless hours manually reviewing patient charts [8,9].

Particularly for back pain, although a very important variable for patient diagnosis and treatment, pain chronicity is often difficult to ascertain from structured EHR fields. Due to the absence of standardized criteria, pain chronicity needs to be manually annotated by clinicians in EHRs, which is not only time consuming but also has the potential to introduce variability in analysis and interpretation among practitioners. Clinical documents, such as clinician notes, diagnosis codes, laboratory reports, procedures, and hospital visits, can be inputted into models using natural language processing (NLP) [10]. NLP with machine learning of clinical documents in the EHRs may enable automated injury scoring [9]. This is a major impediment to back pain research because the duration of symptoms plays a crucial role in predicting the progression of back pain over time.

The automation of data extraction processes from EHRs can significantly alleviate the burden on clinicians, who would otherwise spend countless hours manually reviewing patient charts [8,9]. We hypothesize that pain chronicity can be automatically extracted from the EHR using statistical and NLP techniques, thus making the information available for research in large EHR databases. These techniques have the potential to extend to larger EHR databases in the future, such as the Observational Health Data Sciences and Informatics network, which includes data from more than 810 million individuals [11].

## Methods

### Study Design

This was an observational cohort study with data retrospectively collected from EHRs of patients who visited a specialty spine clinic, the Integrated Spine Services at the University of California, San Francisco, from December 2017 to March 2020. The Integrated Spine Service is a multidisciplinary program focused on improving the quality of care delivered to patients with spinal (cervical, thoracic, or lumbar) pain [12]. Our dataset consisted of outpatient EHRs, which included information on social determinants of health and clinical notes.

The study included patients aged 25 to 97 years with chronic back pain who had been diagnosed by a clinician using an *International Classification of Diseases, 10th Revision* (ICD-10) code ranging between M54.0 and M54.9 [13], had a documented history of pain lasting more than 3 months, and had complete EHRs, including social determinants of health information. Patients were excluded if their files were not annotated with “chronic back pain” or the corresponding ICD-10 code or if their EHR was incomplete or missing. The overall goal of the study was to examine the relationship between expert-annotated pain chronicity and various factors and to evaluate the feasibility of automating the annotation of pain in the EHR of patients with chronic back pain.

### Ethical Considerations

This study received institutional review board approval from the University of California, San Francisco (20-31733). The institutional review board determined that the study qualifies as non–human subjects research, as it involved a retrospective analysis of fully deidentified EHR data collected as part of a clinical quality initiative. Therefore, informed consent was not required. All patient data used in this study were deidentified before analysis and are available upon institutional review. Identifiers such as names, dates, addresses, and medical record numbers were removed in accordance with the Health Insurance Portability and Accountability Act (HIPAA) Safe Harbor guidelines and institutional policy. The research team did not have access to the key linking codes to reidentify the data. There was no direct interaction with participants, and no patients were contacted or compensated for the purposes of this study. No identifiable images or other personally identifying information were included in the manuscript.

### Primary Outcome and Features

The primary outcome of interest was pain chronicity, which was assessed by its duration in months and recorded by clinicians. Pain chronicity was manually annotated by clinicians based on the duration of chronic back pain (Multimedia Appendix 1). The scale ranged from 0 months to decades, which was determined by clinicians based on their assessment of the EHRs. For modeling, chronicity was capped at 66 months (≥5.5 years), as this was determined to be sufficient for research or predictive modeling and matched the maximum amount of time available in the EHR. This information was captured retrospectively from the electronic medical records by reviewing the ICD-10 codes assigned during visits and clinical notes. ICD-10 codes ranging from M54 to M54.9 were used, as each value corresponds to a specific diagnosis. Features that were consolidated into the model were retrospectively collected from the EHR system. The data included age (years), sex (male or female), clinical notes (physician observations and diagnoses), BMI (kg/m^2^), height (cm), race, ethnicity, insurance details, smoking status, first visit date, last visit date, STarT Back risk level, Patient-Reported Outcomes Measurement Information System scores, Area Deprivation Index information, hospital visits to the neurology department, hospital visits to the orthopedic department, and imaging tests (eg, magnetic resonance imaging, computed tomography, and x-ray). These features were included, as they were determined to be relevant to the incidence of chronic back pain based on clinical judgment and previous research.

### Statistical Analysis

RStudio (version 2023.09.1; RStudio PBC) was used for all statistical analyses. Our aim is to explore the relationship between pain-related diagnosis and patient characteristics. To understand the sample, we conducted univariate regression analysis to explore the relationship between annotated pain chronicity—our dependent variable—and a range of potential predictors. These predictors encompassed demographic factors, such as age, sex, and race and ethnicity, as well as clinical indicators, including anxiety; depression; and various medical conditions such as heart disease, high blood pressure, and diabetes. In addition, lifestyle factors such as smoking status and opioid use were considered. The analysis also incorporated the presence of specific ailments such as asthma, migraine, and rheumatoid arthritis, among others. All features were included in the model, and their associations with the outcome were evaluated based on their respective estimates, SEs, test statistics, and *P* values.

### Natural Language Processing

Our study Our study used Apache cTAKES (Clinical Text Analysis and Knowledge Extraction System), which is a powerful open-source NLP model. The cTAKES builds on existing open-source technologies—the Unstructured Information Management Architecture framework and OpenNLP NLP toolkit. Its components, specifically trained for the clinical domain, create rich linguistic and semantic annotations [14]. After processing the clinical notes using cTAKES, the extracted diagnosis codes can be used in the same manner as the structured diagnosis codes.

### Machine Learning Model

The caret package in R (R Foundation for Statistical Computing) was used to train a random forest model [15]. Pain chronicity was used as the outcome variable, and predictors were constructed using structured and unstructured EHR data collected before the last visit date. To train the model, performed a 10-fold cross-validation with 1 repetition. To compare, we trained one random forest model using only structured data and another model using both structured data and NLP results from the unstructured clinical notes. Only NLP-extracted information related to pain diagnosis codes was used and included in the “pain counts before last visit” variable. We used the Pearson correlation coefficient and the mean absolute error (MAE) to assess model performance.

## Results

Our cohort consisted of 1295 patients with orthopedic pain. Within this cohort, our extracted sample size included 394 patients who met criteria for specific variables of interest, including, but not limited to, diagnosis codes (ICD-10: M54.0 to M54.9), free-text notes, patient visit history, and imaging tests related to chronic back pain. Before conducting a demographic analysis specific to our sample size, we conducted a manual check and found 8 instances where the first visit date of the patient was missing.

Thus, our final sample included 386 patients who were residents of the San Francisco Bay Area, with 70.2% (n=271) residing in urban areas, 12.4% (n=48) in predominantly suburban areas, 10.6% (n=41) in upscale suburban areas, and 3.9% (n=15) in the San Francisco Peninsula. The median age of the sample was 62.0 (IQR 48.8-72.0) years, and the mean age was 60.2 (SD 16.1) years. Our sample comprised 62.7% (242/386) of patients who identified as women and 37.3% (144/386) who identified as men. Race was predominantly defined by 3 categories (ie, 189/386, 49% White; 85/386, 22% Asian; 56/386, 14.5% Black or African American), and 9.1% (35/386) of the patients identified as Hispanic or Latinx. The sample had 3 insurances: Medicare (125/386, 32.4%), commercial insurance (37/386, 9.6%) and Medi-Cal Managed care (31/386, 8%; Table 1).

**Table 1:** Demographic characteristics of patients with chronic back pain, identified by International Classification of Diseases, 10th Revision, codes in the electronic health records, who visited the Interdisciplinary Spine Clinic at the University of California, San Francisco, between December 2017 and March 2020 (N=386).

Variable			Values, n (%)
**Sex**			
	Female	242 (62.7)	
	Male	144 (37.3)	
**Race**			
	American Indian or Alaska Native	1 (0.3)	
	Asian	85 (22)	
	Black or African American	56 (14.5)	
	Native Hawaiian or other Pacific Islander	4 (1)	
	Other Pacific Islander	1 (0.3)	
	White	189 (49)	
	Other	44 (11.4)	
	Declined	6 (1.6)	
**Ethnicity**			
	Hispanic or Latino	35 (9.1)	
	Not Hispanic or Latino	342 (88.6)	
	Declined	8 (2.1)	
	Unknown	1 (0.3)	
**Bay Area region**			
	San Francisco city	271 (70.2)	
	East Bay	48 (12.4)	
	North Bay	41 (10.6)	
	San Francisco Peninsula	15 (3.9)	
	Missing	11 (2.8)	

The information in the EHRs from the spine clinic consisted of multiple variables. First, we conducted a univariate regression analysis to ascertain each variable’s individual association with chronic pain severity. The analysis indicated a positive relationship between the pain severity before the last visit, the number of prior imaging orders (*P*<.001), the number of prior visits to the neurology department (*P*=.01), Medi-Cal insurance (*P*=.02), prior opioid antagonist prescriptions (*P*=.08), and a negative relationship with commercial insurance (*P*=.05). Table 2, ordered by ascending *P* values, highlights the most influential predictors of chronic pain severity based on their association strength.

**Table 2:** Top 10 variables from the structured electronic health records associated with manually annotated pain chronicity levels in patients with chronic back pain who visited the integrated spine clinic at the University of California, San Francisco, between December 2017 and March 2020.

Variable	Estimate (SE)	Coefficient	*P* value
Pain counts before the last visit	6.2289928 (1.2429963)	5.0112722	<.001
Number of prior imaging orders (MRI^a^, CT^b^, or X-ray)	0.4973738 (0.1671713)	2.9752338	.003
Number of prior visits to the neurology department (spine)	0.6731830 (0.2472161)	2.7230548	.007
Insurance type (Medi-Cal)	7.8438835 (3.8850542)	2.0189895	.04
BMI	0.4714030 (0.2693194)	1.7503493	.08
Number of prior visits to the orthopedic department	0.2661974 (0.1650582)	1.6127484	.11
All clinical notes	0.0635035 (0.0462438)	1.3732308	.17
ADI^c^ rank (state)	1.3598764 (1.0230850)	1.3291920	.18
Number of prior visits to the neurosurgery department	0.6092814 (0.4744465)	1.2841941	.19
Number of hospital or ED^d^ visits	−0.2283034 (0.2096801)	−1.0888178	.28

^a^MRI: magnetic resonance imaging.

^b^CT: computed tomography.

^c^ADI: area deprivation index.

^d^ED: emergency department.

**Figure 1:**
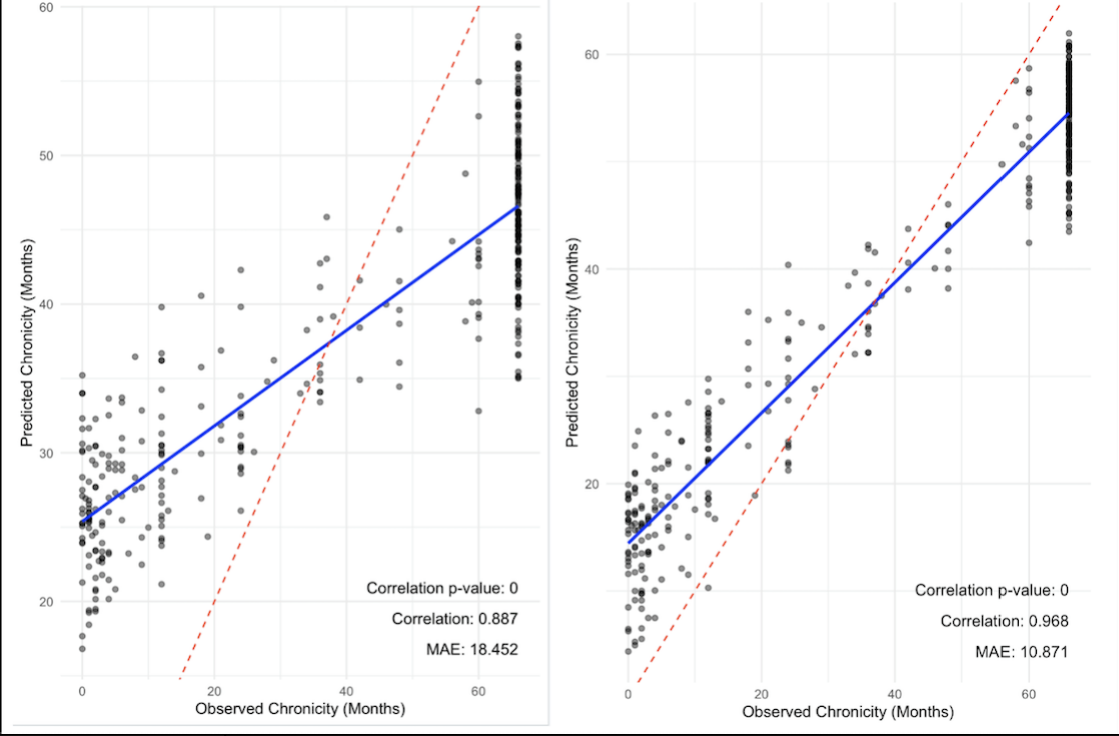
(A) Accuracy of prediction of pain chronicity levels using structured electronic health record data without utilization of natural language processing tools. (B) Accuracy of prediction of pain chronicity levels using unstructured electronic health record data with utilization of natural language processing tool. MAE: mean absolute error.

Univariate regressions were performed to assess the association of structured EHR variables with the manually annotated pain chronicity. The results are sorted by *P* value, and only the top 10 variables are included. Next, we used the random forest machine learning model to assess the performance of extracting pain chronicity from the EHR. For the model using only structured data (Figure 1A), a strong correlation was observed between predicted and observed chronicity values, with a correlation coefficient of 0.887 and an MAE of 18.45 (correlation *P* value =0).

**Figure 2:**
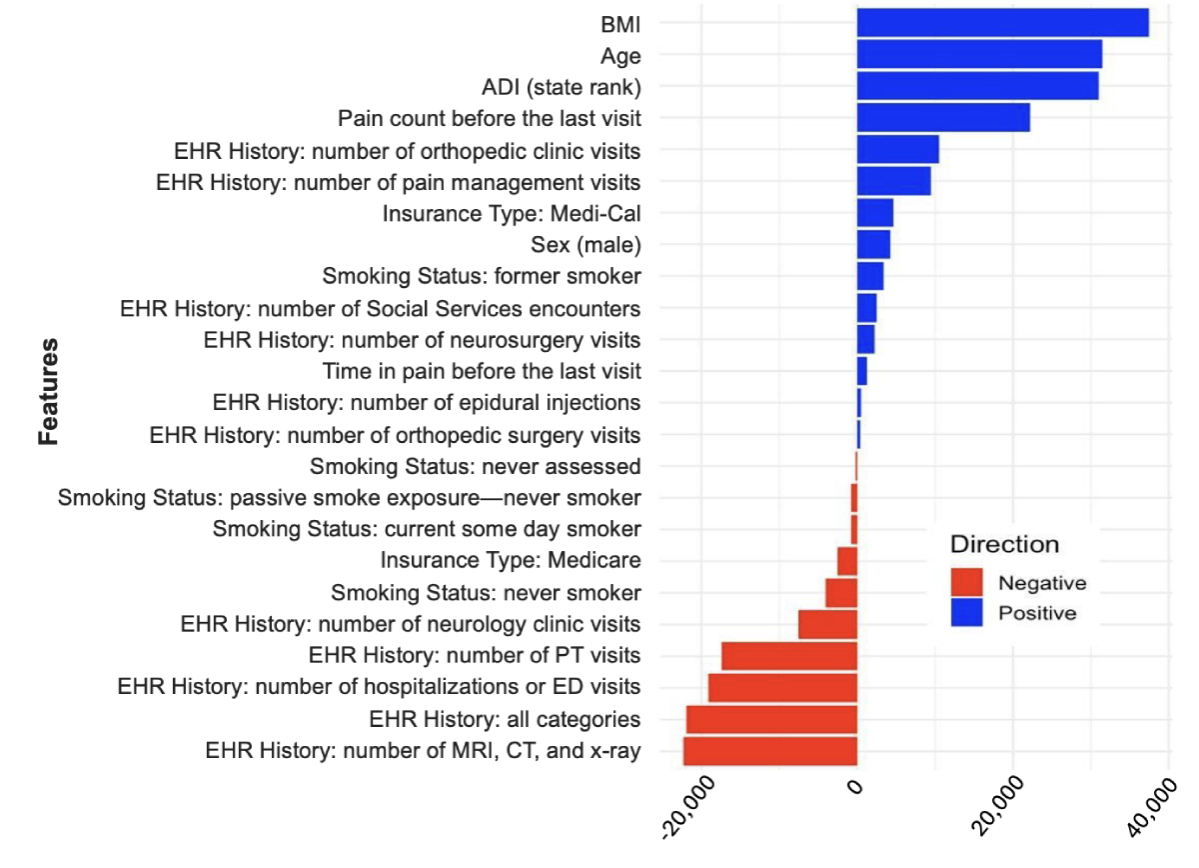
Feature importance ranked by mean decrease in Gini from a random forest model, which is predicting pain chronicity levels from electronic health records of patients with chronic back pain. ADI: area deprivation index; CT: computed tomography; EHR: electronic health record; MRI: magnetic resonance imaging; PT: physiotherapy.

However, when using NLP tools to extract information from unstructured data (Figure 1B), the correlation coefficient increased to 0.968, with an MAE of 10.87. This suggested improved accuracy in predicting chronicity when using NLP tools compared to the model without NLP tools. Both approaches demonstrated strong correlations (structured model: *r*=0.887, *P*<.001; NLP-enhanced model: *r*=0.968, *P*<.001) between predicted and observed chronicity values, with predictions showing a lower MAE. The use of NLP tools resulted in more accurate predictions with lower MAE. Overall, the most important features for predicting pain chronicity in the NLP model (Figure 2) were BMI, age, area deprivation index, and pain count before the last visit predicted by the model to increase the chronicity of pain, whereas EHR visit history and smoking status (never smoker) were used by the model to predict decreased pain chronicity.

## Discussion

### Principal Findings

Our study successfully demonstrated the feasibility of automatic extraction of pain chronicity from EHRs using a combination of structured data and NLP techniques on unstructured clinical notes. Our findings support our initial hypothesis that automation of this process is feasible, which reduces the need for manual chart review by clinicians. Our results highlight the potential for application of this method to larger EHR databases, which would enable expansive chronic pain research. The initial univariate regression analysis identified several variables significantly associated with chronic pain severity. A positive relationship was observed with the number of pain diagnoses before the last visit, prior imaging orders, prior visits to the neurology department, Medi-Cal insurance, and prior opioid antagonist prescriptions, while a negative relationship was noted with commercial insurance. Furthermore, we used a random forest machine learning model and used an NLP tool to compare the accuracy between the use of just structured EHR data versus the use of structured EHR data and the information extracted from unstructured clinical notes with the help of the cTAKES tool. As evidenced by a lower MAE of 10.87, the model that used both structured data and unstructured information extracted by the NLP tool had superior performance.

The use of EHRs to develop a predictive model for lower back pain outcomes is not a novel concept. In an observational study to identify acute low back pain in primary care practice from clinical notes [16], the researchers noticed that due to a lack of distinguishing codes, both chronic and acute low back pain are coded in the EHRs with the same ICD-10 code and can only be differentiated by retrospective chart reviews. The objective of their study was to evaluate the feasibility of automatically distinguishing acute low back pain by analyzing free-text clinical notes. A dataset of 17,409 clinical notes from different primary care practices was used, of which 891 documents were manually annotated as acute low back pain and 2,973 were generally associated with low back pain based on the recorded ICD-10 code.

Published studies have reported that the use of machine learning models on EHR data has been successful in the extraction of predictors of pain. In a retrospective study that used EHR data from UConn Health John Dempsey Hospital applied NLP technology to an EHR dataset as part of a pilot study to capture pain information from clinical notes and prove its feasibility as an efficient method. Their pain extraction model created using the NLP tool was successful in extracting granular pain parameters from the clinical notes. The model identified back pain as the most commonly reported pain location, with 40,369 term frequencies. In identifying body location, the model has a precision value of 0.78 and a recall value of 1, resulting in an *F* score of 0.87. In identifying co-occurring symptoms within the clinical note, the model has a precision value of 1 and a recall value of 1, resulting in an *F* score of 1 [17].

Different supervised and unsupervised strategies were used for automated identification, such as keyword search, topic modeling, logistic regression with bag-of-n-grams and manual features, and deep learning models (a convolutional neural network [ConvNet]–based architecture). They trained the models using either manual annotations or ICD-10 codes as positive labels. The results showed that the ConvNet trained using manual annotations obtained the best results, with an area under the receiver operating characteristic curve of 0.98 and an *F* score of 0.70. ConvNet’s results were also robust to reductions in the number of manually annotated documents. It was also seen that in the absence of manual annotations, topic models performed better than methods trained using ICD-10 codes, which were unsatisfactory for identifying low back pain acuity [16]. This study uses clinical notes to analyze and understand different therapeutic treatments, guidelines for billing, and management options for acute low back pain. Similarly, we leveraged information in EHRs to automate the extraction of pain chronicity and used both machine learning and NLP methods.

### Limitations

We worked with retrospective data, which come with their own drawbacks, such as conscious or unconscious bias and lack of control toward confounding factors. Due to this, assessment of chronicity of back pain was subjected to biased interpretation of symptoms by clinicians based on their experiences and the subjective nature of patient reporting, which can often be influenced by health literacy and social determinants, potentially leading to misdiagnosis. Our sample size is left skewed, with most of the patients having long-standing back pain (ie, for ≥5 years), which makes generalization of our results difficult. As our data source is from the EHR, there is a high likelihood of incomplete notes, which poses a significant challenge because accurate information is required to provide successful patient care. This issue is highlighted when essential information is missing, which can hamper the accuracy of our model. Communication barriers resulting in misinterpretation of patient conditions, redundant tests, and absence of visit dates are some of the common gaps that can compromise patient safety, which may be misinterpreted by the machine learning model, resulting in inaccurate results.

Defining chronic pain (chronicity) is also a challenge because there is an absence of a precise definition due to the complexity of its nature. In this study, we classified it as an ailment lasting beyond a certain time frame, with a minimum duration of 3 months.

### Future Research

The observations of our study strongly suggest that the analysis of pain chronicity using EHR data holds promise for exploring a broader range of topics on larger datasets in the future, eliminating the necessity for manual annotation.

### Conclusions

By leveraging the information in EHRs using machine learning models, which contain information on patient health over time, we can potentially delve into various aspects related to chronic pain without the labor-intensive process of manual annotation. This approach not only offers adaptability but also opens doors to uncovering insights into the progression, management, and impact of chronic pain on individuals and populations. In addition, the use of larger datasets can enhance statistical power and allow for more robust analyses, enabling researchers to explore nuanced relationships and trends related to chronic pain with greater accuracy and confidence. Consequently, our study underscores the potential of leveraging clinical data to advance our understanding of pain chronicity and its broader implications for health care and research, which can pave the way for future investigations into this critical area without the constraints of manual annotation.

## Appendix

Multimedia Appendix 1 Frequency distribution of manually annotated pain chronicity (in months) by clinicians in the electronic health records (EHRs) of patients was collected at the interdisciplinary spine clinic at University of California, San Francisco, between December 2017 and March 2020. The study population included patients from the San Francisco Bay Area aged between 25 and 97 years. Pain chronicity levels were manually annotated through EHR review, with a focus on the number of visits in this retrospective observational study.

## Abbreviations

cTAKES: clinical Text Analysis and Knowledge Extraction System
EHR: electronic health record
HIPAA: Health Insurance Portability and Accountability Act
ICD-10: International Classification of Diseases, 10th Revision
MAE: mean absolute error
NLP: natural language processing

## Data Availability

The data analyzed in this study were derived from electronic health records and contain protected health information. For this reason, the data are not publicly available. Deidentified data may be made available from the corresponding author on reasonable request, subject to approval by the appropriate institutional review board and in accordance with institutional policies and applicable data use agreements.
