# Supplementary figures and images for: Automated Annotation of Pain Chronicity in Patients With Back Pain by Using Electronic Health Records: Retrospective Study
Source: JMIR Form Res. 2026 Mar 5;10:e63198. doi: 10.2196/63198 (PMC13003205; doi:10.2196/63198)

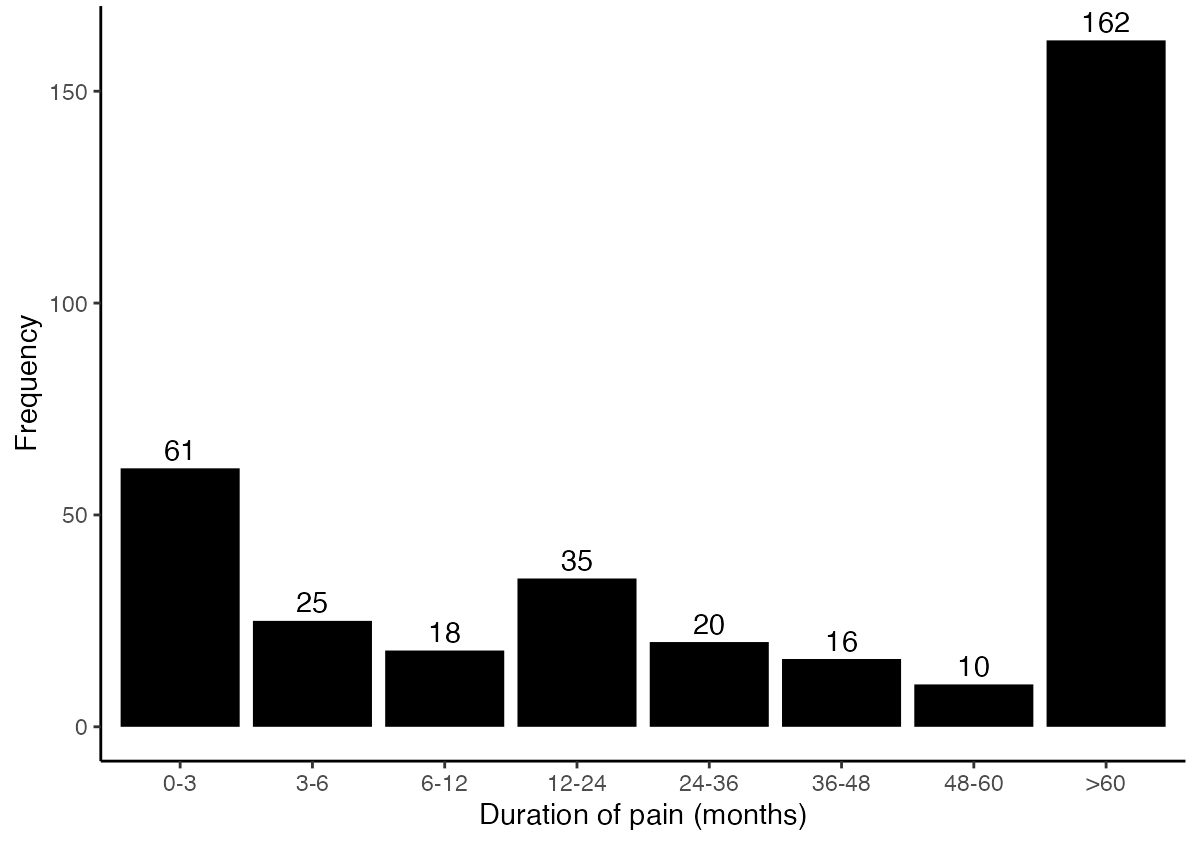

Supplement: Multimedia Appendix 1 [file formative_v10i1e63198_app1.png]
